# Use of Actigraphy for a Rat Behavioural Sleep Study

**DOI:** 10.3390/clockssleep3030028

**Published:** 2021-08-02

**Authors:** Shinichi Esaki, Meiho Nakayama, Sachie Arima, Shintaro Sato

**Affiliations:** 1 Department of Otolaryngology, Head and Neck Surgery, Nagoya City University Graduate School of Medical Sciences and Medical School, Nagoya 467-8601, Japan; sesaki@med.nagoya-cu.ac.jp (S.E.); mnakayama@suimin-memai.com (M.N.); sachie_arima@yahoo.co.jp (S.A.); 2 Good Sleep Center, Nagoya City University Hospital, Nagoya 467-8602, Japan; 3 Department of Virology, Graduate School of Medicine, Nagoya University, Nagoya 464-8550, Japan; 4 Meiho Sleep & Balance Clinic, Nagoya 450-0002, Japan

**Keywords:** insomnia, behaviour, animal, actigraph

## Abstract

Previous studies of animal behavioural sleep is mainly divided into two study types, observation by video recording or counts by sensor, both of which require a complex environment and procedure. An actigraph unit is a commercially available product which can provide non-invasive monitoring human rest/activity cycles. The goal of this study was to evaluate whether actigraphy can be applied for analysing behavioural sleep in rats, since no reports have described utilization of the actigraphy unit for monitoring sleep of small animals. The actigraph unit was held on the chest of eight male rats by a loose elastic belt. The rats spent two days in a normal condition, followed by two days of sleep deprivation. Total counts measured by the actigraph could be clearly divided into two phases, sleep phase and awake phase, when the rats were kept in the normal cage. Next, the rats were moved into the sleep-deviation cage, and the total counts were significantly higher during daytime, indicating the successful induction of sleep deprivation. These results showed that the actigraphy unit monitored rest/activity cycles of rats, which will contribute to making sleep behaviour experiments easier.

## 1. Introduction

Sleep is an essential component of health, with its timing, duration, and quality being critical determinants of health [1,2,3]. Animal experiments have shown that impaired sleep affects metabolic and emotional regulation, performance, memory consolidation, brain recuperation processes, and learning [4,5,6,7,8,9]. Animal experiments on sleep research have been classified as either: behavioural sleep—quantified by behavioural observations [10,11,12,13,14], or electrographic sleep—quantified by electrographic recordings [15,16,17,18,19]. The latter is less frequently studied over the former as it requires complex preparation and analysis of electrographic recordings. Studies of animal behavioural sleep are mainly divided into two study types: observation by video recording or counts by sensor, such as an infrared photocell [10,11,12,14]. For such studies, researchers had to create a particular environment for the experiment, and they had to concentrate on counting, observing, and analysing animal behaviour.

The actigraph unit (or actimetry sensor) is commercially available and provides a non-invasive method of monitoring human rest/activity cycles. Gross motor activity is continually measured by an actigraph in a wristwatch-like device. Data from such can be transferred to a computer and easily analysed offline [13,20,21,22,23,24]. As commercially available actigraph units are created for humans, several animal sleep experiments have employed such to study large animals, such as monkeys [18], dogs [25], or horses [26]; but not for small animals, such as rats or mice. However, animal sleep experiments are frequently performed on these small animals [10,14,27,28,29,30]. The goal of this study was to investigate whether actigraphy can be applied for the analysis of rats.

## 2. Results

### 2.1. Activity under Control Condition

**Figure 1:**
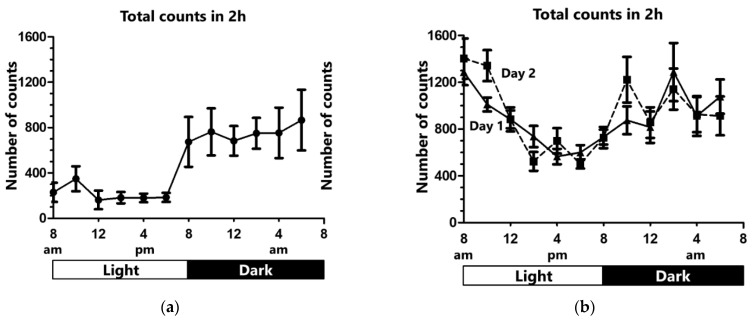
Total ‘counts’ measured by the actigraph unit. (**a**) Under the control condition, total counts were lower during the light cycle, and higher during the dark cycle, indicating a sleep phase in the light and an awake phase in the dark. (**b**) In the sleep deprivation cage, the activity of the rats was higher compared with the control condition. Total counts did not differ significantly between day 1 and day 2. Data are shown as mean ± standard error of means.

**Figure 2:**
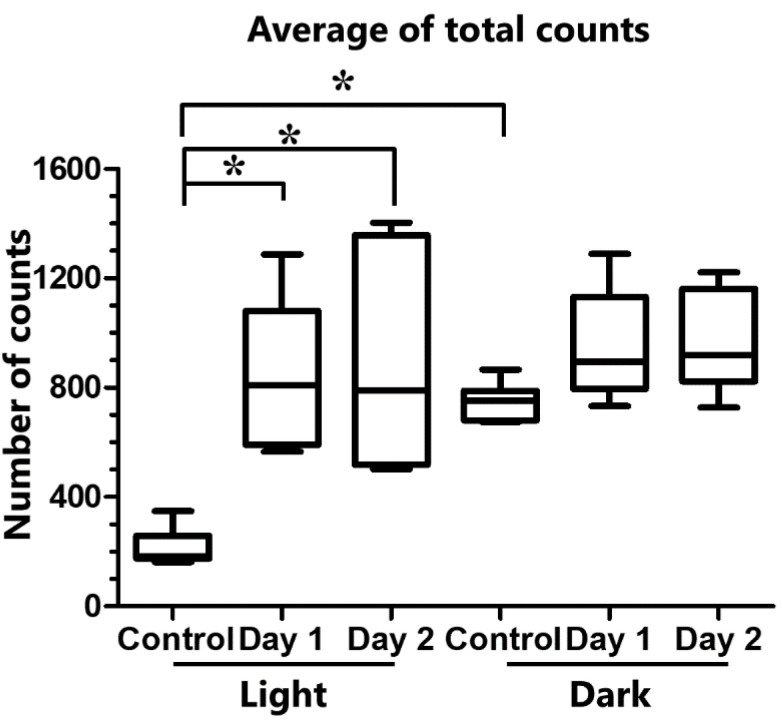
The 2-h total count average. Under the control condition, total count average was higher in the dark cycle compared with such in the light. In the light, the count average was significantly higher for day 1 and day 2 under sleep deviation compared with control conditions (* *p* < 0.05). In the dark, the count average was slightly higher under sleep deviation, which was not significant compared with control. Data are shown as mean ± standard error of means.

The average of total counts in each two-hour period were plotted in Figure 1a. Total number of counts showed a natural division into two cycles: total counts below 400 in the light, and total counts above 400 in the dark. The average of total counts was 214.8 ± 94.0 in the light and 748.4 ± 202.6 in the dark, suggesting a sleep phase in the light and an awake phase in the dark (Figure 2). There was a significant difference in total counts between these phases.

### 2.2. Activity under Sleep Deprivation

The average total counts in each two-hour period in the first 24 h (day 1) and in the second 24 h (day 2) are plotted in Figure 1b. No significant difference was observed in the total counts of 2 h between day 1 and day 2. However, the average of total counts in the light was significantly increased in day 1 and day 2 compared with the control (Figure 2), indicating more activity due to the sleep-deviation cage. The activity also seemed higher in the dark in day 1 and day 2; however, no significant difference was observed when compared with the control condition.

## 3. Discussion

The assessment of locomotor and exploratory behaviour in rodents is one of the most widely used behavioural methods to determine the effects of genetic, physiological, and pharmacological manipulations for humans. An extensive variety of measures have been used for behavioural sleep to quantify motor activity in rodents. The oldest and most common paradigm is the open field test [31,32]. A small rodent is placed in the field for a fixed time interval and a number of activity variables are quantified, including the distance covered per unit time, the number of regions visited, rearing, latency to initial movement, stereotypic behaviours (e.g., sniffing or grooming), and physical responses (e.g., defecation or urination). To simplify the open field test, a container with the floor marked into discrete regions was developed, and an observer manually scored rodent transitions between the regions and quantified entries. Since rodents are nocturnal, observation itself may affect their behaviour. To alleviate problems associated with direct observation of rodents and to simplify data collection, a number of automated open field instruments have been developed, including photobeam and video recording systems [33,34,35,36].

As shown above, previous behaviour studies required a rather large environment, and a complex means of observation [10,11,12,14]. Furthermore, results can be affected by many factors, including their condition, illumination, ambient noise, and time. Therefore, it was difficult to quantify behaviour and to obtain reliable results. Actigraphy is designed for humans to show their activity and sleep. The present study is the first to directly measure rat activity and behaviour via actigraphy, and to precisely quantify their movement.

From the first experiment under the control condition, the rats’ activity was shown by the actigraph as clearly divided into two phases: the sleep phase in the light, and the awake phase in the dark. Following, the rats were placed in a special cage for sleep deprivation (which is frequently used in research to evaluate the hypnotic effect [28,37]). Increased activity through the day indicated successful induction of sleep deprivation. Actigraphy proved a useful tool for the investigation of sleep behaviour without harming the rats’ life cycle.

Sleep disturbance may be a risk factor for the development of the ADHD, or a symptom or a comorbid condition of ADHD, affected by similar psychopathology [38,39,40]. Sleep deprivation has become a major problem not only affecting human health but also social and development defects. Unfortunately, actigraphy of rats’ sleep behaviours and animal experiments studying sleep behaviour regarding these problems are still insufficient. We hope this study will facilitate easier experimentation and encourage more researchers to undertake experiments in this area to elucidate sleep disorders, which are on the rise.

## 4. Materials and Methods

### 4.1. Actigraphy

The actigraph device, the GT3X from ActiGraph (Pensacola, FL, USA) was utilised and data analysed via ActiLife (version 6) data analysis software from the same company. The GT3X provides objective 24-h physical activity and sleep/wake assessment by monitoring activity with either the ‘threshold crossing’ or ‘cycle count’ method; activity is shown by ‘count.’ The threshold crossing technique involves incrementing a ‘count’ each time the magnitude of acceleration (activity) exceeds a given threshold. The cycle count technique produces a ‘count’ when sufficient force is applied to move a mechanical lever through a full cycle (up and down). These two techniques are very similar in nature to that of the modern-day pedometer.

### 4.2. Animals

Eight adult male Wistar rats (Japan SLC Inc., Shizuoka, Japan) weighing between 300 and 350 g were used. All animals were maintained in an air-conditioned room with controlled temperature (24 ± 2 °C) and humidity (55 ± 15%). They were housed in aluminium cages with sawdust flooring and kept under a light (200 lux)–dark (5 lux; LD) cycle (lights on from 8:00 am to 8:00 pm). The animals were allowed free access to food and water before and during the sleep deprivation. These procedures were conducted in accordance with the approval of the Animal Ethics Committee of Nagoya City University, Graduate School of Medical Sciences.

### 4.3. Sleep Deprivation Cages

To create sleep deprivation, each rat was placed in a specially designed test cage [28,30]. The cage was a plastic cylinder (diameter, 26 cm; height, 31 cm), with its floor covered by a grid of stainless-steel rods (3 mm wide; 2 cm apart). It was filled with water up to 2 cm below the grid surface. The rats were thus subjected to two relatively powerful stressors: the grid and water. Hence, the arousal time of rats placed on the grid was longer than that of rats placed on sawdust. Sleep deprivation occurs while rats are under these conditions, as confirmed by electroencephalogram recording; rats on the grid had significant increases in sleep latency and duration of wakefulness observed compared with rats on sawdust [41].

### 4.4. Activity under Control Conditions and Sleep Deprivation

**Figure 3:**
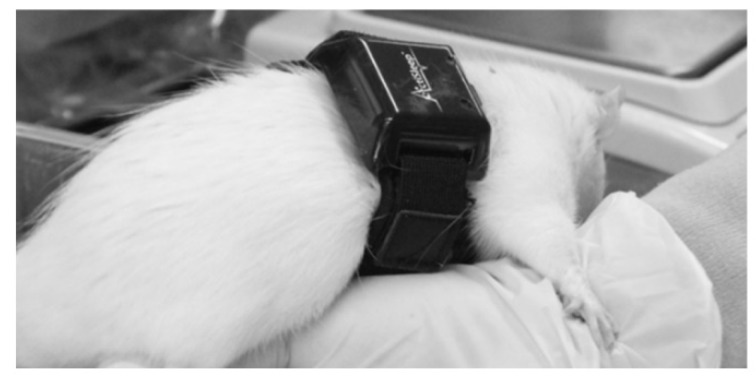
A representative picture of rat wearing the actigraph around its chest area.

As shown in Figure 3, the actigraph was strapped to the rat with a loose elastic belt, to avoid disturbing its breathing. The rats (*n* = 8) wore the actigraph under anaesthesia with pentobarbital sodium (35 mg/kg; Abbott Laboratories, North Chicago, IL, USA) in the morning of the first day. Data collection started from 8 am the following morning to avoid effects from anaesthesia and were continued for 24 h. Thereafter, each rat was transferred to the sleep deprivation cage, and data were collected over another 24 h. The average total counts of the rats in the normal cage (control) were compared to those of rats in the sleep deviation cage for both periods (day 1 and 2).

### 4.5. Statistics

Data are shown as mean ± standard error of means and were analysed using one-way analysis of variance followed by the Tukey test for multiple comparisons among groups by JMP for Windows version 9 (SAS Institute Inc., Cary, NC, USA). A value of *p* < 0.05 was considered statistically significant.

## Data Availability

Data is contained within the article.
